# Solar maculopathy: prognosis over one year follow up

**DOI:** 10.1186/s12886-019-1199-6

**Published:** 2019-09-18

**Authors:** Marwa Mahmoud Abdellah, Engy Mohammed Mostafa, Mohamed Abdelatif Anber, Islam Saad El Saman, Mohammed Ezz Eldawla

**Affiliations:** Ophthalmology Department, Sohag University, Sohag, 82524 Egypt

**Keywords:** Solar maculopathy, Outer retinal hole, Eclipse maculopathy, Foveal lesions from sun rays

## Abstract

**Background:**

To document the visual acuity, spectral domain optical coherence tomography (SD-OCT) findings and prognosis in10 eyes of 6 patients with foveal damage from solar retinopathy in 1 year.

**Methods:**

This was a prospective, observational case series of patients presented by solar maculopathy at Ophthalmology department, Sohag University. All patients underwent visual acuity (VA) testing, refraction, dilated fundus examination fluorescein angiography (FA) and SD-OCT (spectral Domain ocular coherence Tomography) imaging and follow up for 1 year.

**Results:**

The mean age was 16.5 years (range 9–27 years, both eyes are affected in 4 patients. The mean spherical equivalent (SE) was – 0.25 ± 0.50 D.

The visual acuity of the affected eyes ranged from 0.4 to 0.9 on presentation. At presentation Significant foveal pathology was identified on SD-OCT in 10 eyes, All eyes showed disruption of the photoreceptor ellipsoid zone and the interdigitation zone on SD-OCT, Follow up of the cases continued for 1 year.100% of cases showed improvement in VA: 20% eyes regained 1, 50% eyes with VA of 0.9; two eyes 20% 0.8 and one eyes (10%) with 0.4. The improvement began after 1 week and reached its maximum and became stationary after the 6th month of follow up, the outer retinal hole persist in OCT in 80% of cases.

**Conclusion:**

Solar maculopathy has a good prognosis yet shows no improvement after 6 months. Young age might pose as a risk factor.

## Background

The harmful effects of sunlight were reported in the form of thermal and photochemical damage in the retinal pigment epithelium and photoreceptors. The histological damage occurs at the level of the retinal pigment epithelium melanosomes and photoreceptor external segment [1].

The main natural defense mechanism against the harmful ultraviolet rays is the cornea and crystalline lens, as they selectively block most of the ultraviolet radiation of 400 nm but allow the transmission of the visible and infrared radiation between 400 nm and 1400 nm to the retina. Photic retinopathy can occur upon prolonged light exposure without protective measures [2]. Solar maculopathies can be caused by a single or also recurrent exposure [3, 4].

The fact that the fovea is not protected by the ganglion layer makes it more liable to be affected by solar radiation [5].

The risk factors for macular affection which were documented before depends mainly on the intensity, duration and range of exposure so many studies claimed that dilated pupils, transparent media, and albinos posed an increased risk for more macular damage [6].

The diagnosis of solar maculopathy was confirmed by OCT changes, but the follow up of the changes were not well documented. The follow up of VA is not well studied before, so our study aims to track changes in VA and macular OCT over 1 year follow up.

## Methods

This study is a prospective observational case series of solar maculopathy patients who presented to the Ophthalmology outpatient clinic at Sohag University Hospital over a period of 2 years from January 2015 to January 2017.

The mean age of patients was 16.5 years old (range 9 to 27 years old). History taking was suggesting a great susceptibility of solar maculopathy: two children were playing as a competition of how long they can tolerate looking to the sun, another young lady followed the sun at early sunrise, one patient was a builder and exposed to a direct sunlight, two cases were soldiers in the army and stood for a long time in heavy sunrays, (presented at separate times from two different places). The inclusion criteria include; a definite history of an acute drop of vision after direct sun exposure.

Exclusion criteria include; previous drug intake, vision problems before the insult, family history of inherited macular dystrophies, a history of ocular trauma, previous intraocular surgery. All patient were subjected to slit lamp examination of anterior segment examination, visual acuity testing (decimal values) either uncorrected or best corrected. IOP measurement, manifest refraction, fundus examination, and detailed fundus evaluation of the retina by indirect ophthalmoscopy, fluorescein angiography, fundus autofluorescence (FAF) and ocular coherence tomography (OCT) by (Optovue RTVue XR Avanti, Optovue Inc., Fremont, CA) of the macula had been done. All procedures performed in studies involving human participants were in accordance with the ethical standards of the Medical Ethics Committee of Sohag University.”

Informed written consent was obtained from all participants or their guardians included in the study. Follow up was done for 1 year to document the progress of BCVA (Best Corrected Visual Acuity) and estimate the duration elapsing from sun exposure untill improvement in visual acuity. Follow up has been done at 1st week, 1st month, 3rd month, 6th month, 9th month and 12th month. OCT imaging was done in the follow-up visits and after improvement was obtained as well.

Statistical analysis: A simple statistical analysis was used by manual calculation regarding the Mean, Standard deviation and the range, and the findings were reported as a percentage.

## Results

Ten eyes of six patients presented to outpatient clinic (4 males and 2 females), with a mean age were 16.5 years (range 9 to 27 years old) and mean BCVA was 0.3 (range 0.2 to 0.8) at presentation. Both eyes were affected in four cases, one eye was affected in two cases, the affected eye was the dominant eye in unilateral cases. The dominant eye was determined the history as it was the preferred eye in photographing by a camera, in shooting by gun and the emmetropic eye in the case of anisometropia. The mean manifest refraction was − 0.25 ± 0.50 spherical equivalents (SE), central scotoma was a major complaint in 100% of patients. Other complaints were metamorphopsia in 4 eyes (40%). The duration of the sun exposure could not be estimated accurately but the entire patient confirmed that exposure did not maintain more than a few minutes.

**Fig. 1:**
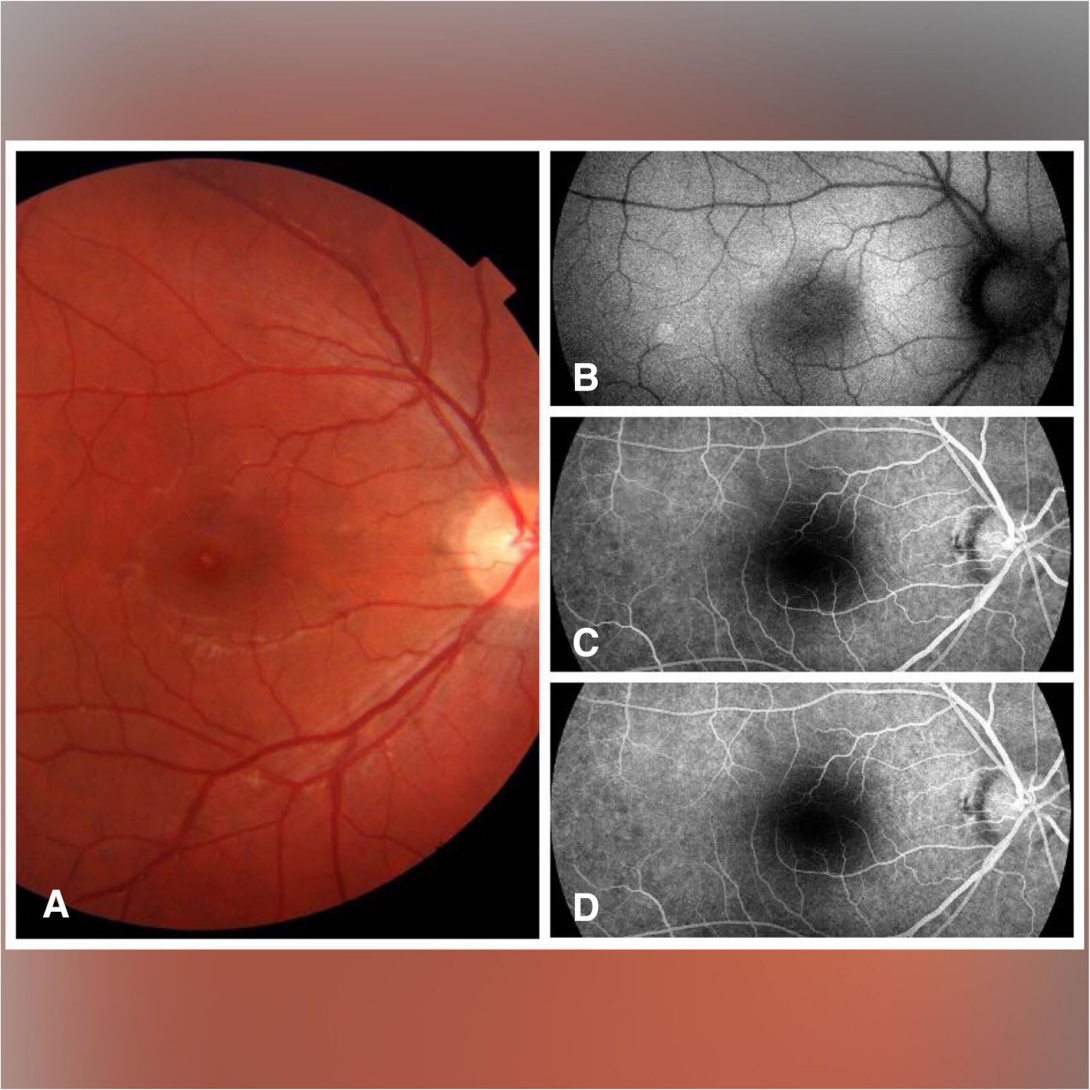
Shows Fundus (**a**), fundus auto fluorescence (**b**) and Fluorescein angiography Early (**c**) and late (**d**) of an affected eye of solar maculopathy in 11 years old showing a whitish spot in the fovea with normal auto fluorescence and with free FA

**Fig. 2:**
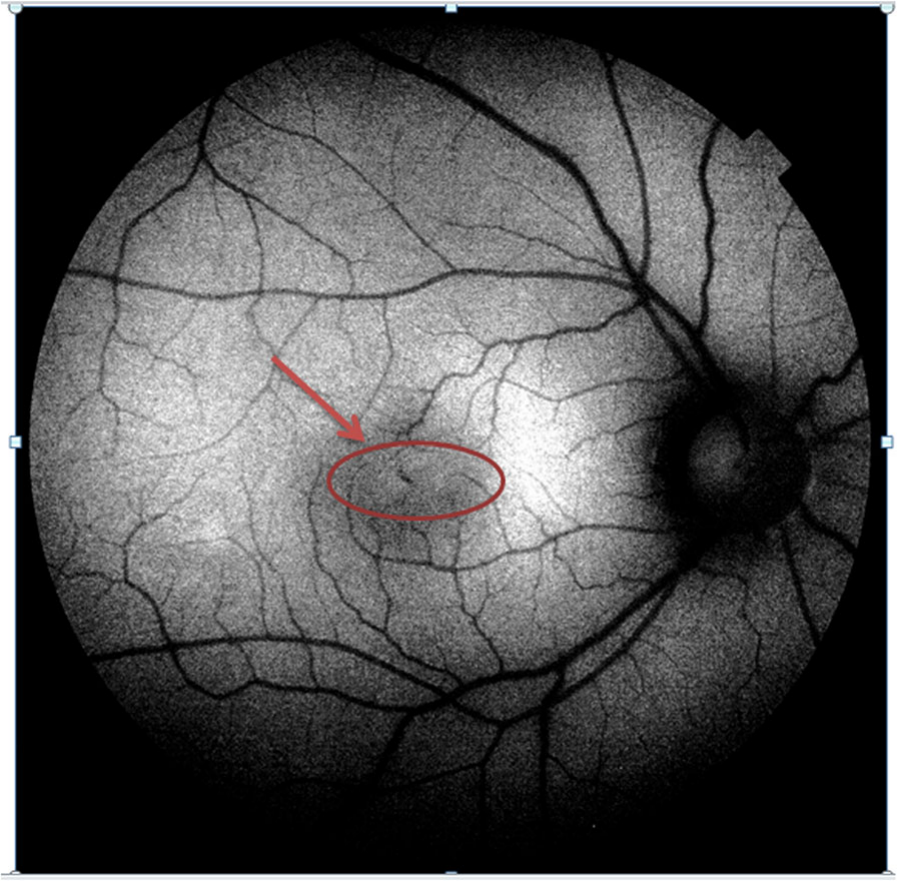
A fundus auto fluorescence image of Rt.eye with solar maculopathy shows a hyporeflective spot in the center of the macula

**Fig. 3:**
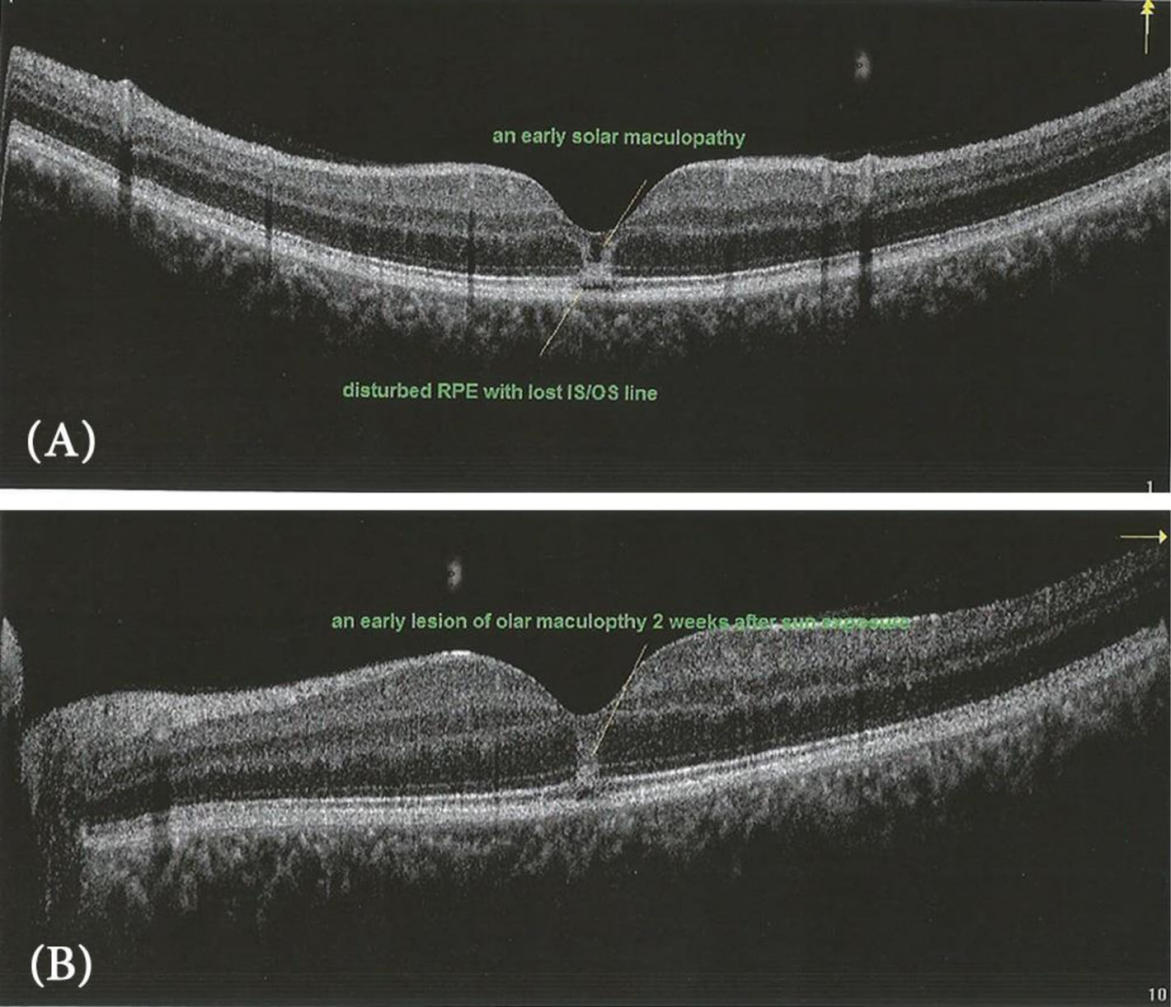
An early picture of solar maculopathy (2 weeks after sun exposure), **a** in the right eye and **b** in the left eye

**Fig. 4:**
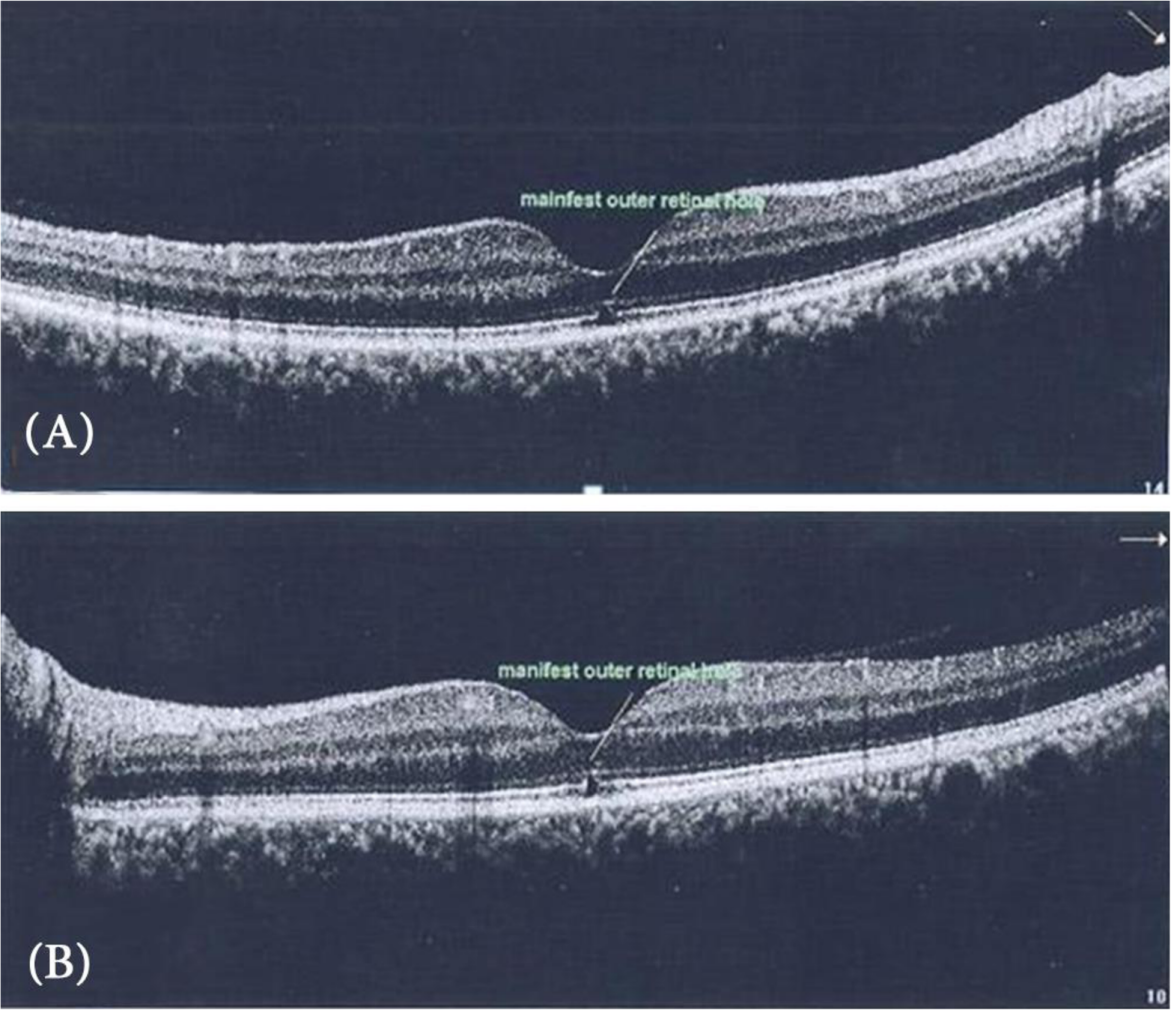
**a**, **b** Shows an outer retinal hole 6 months after sun exposure in the right eye and the left eye respectively

**Fig. 5:**
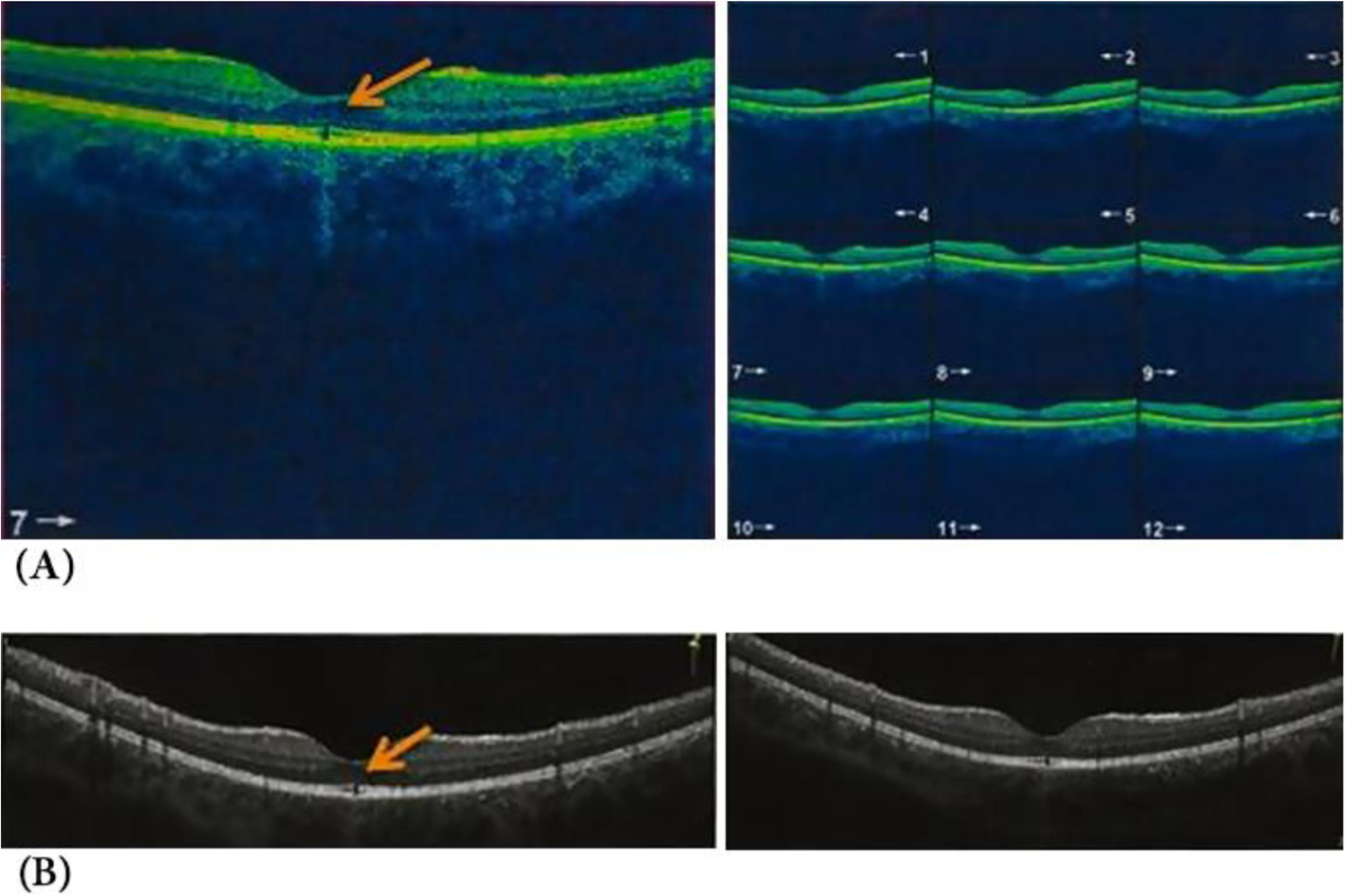
**a** Shows an outer retinal hole with loss of integrity of photoreceptor and IS/OS line in a child of 11 years old. Three days after sun rays exposure. **b** Shows the same pathology after 1 year follow up of the same patient

**Fig. 6:**
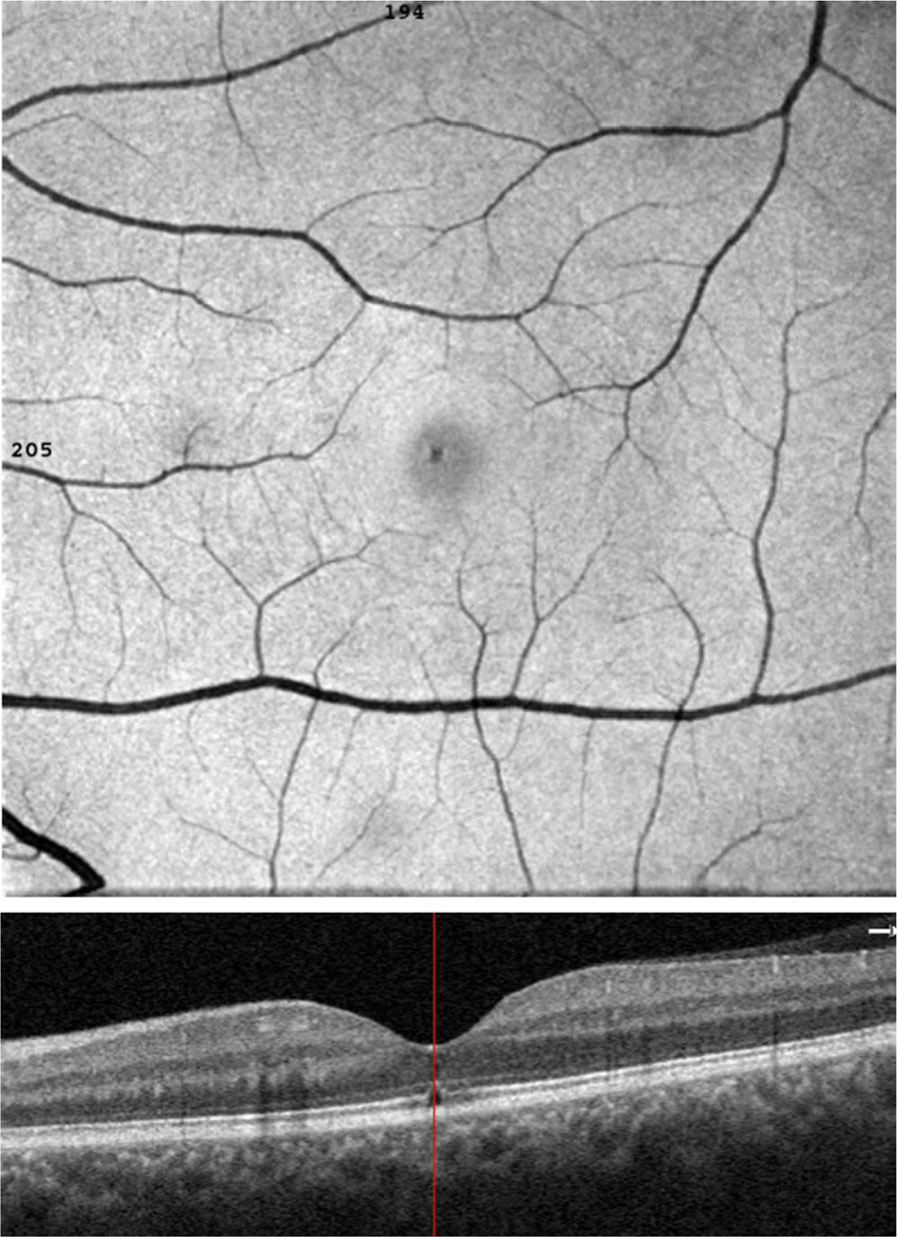
An En Face of outer retina slap data showing a manifest outer retinal hole

The time of presentation ranged from 3 days to 1 week after exposure. Amsler grid was significant for metamorphopsia in 4 eyes 40%, and central scotoma in 8 eyes 80% Anterior segment examination showed no abnormality, normal intraocular pressure in all cases, the fundus picture showed white foveal spots in 4 eyes (40%) with absence of foveal reflex in 7 patients (70%), Fluorescein angiography was free in all cases as shown in Fig. 1, Fundus autofluorescence examinations shows small hyporeflective spot in the fovea in 4 eyes (40%) (Fig. 2), High definition SD-OCT (Optovue RTVue XR Avanti, Optovue Inc., Fremont, CA) showed a focal foveal outer retinal hole at the junction of inner and outer photoreceptor segment in the OCT in 10 eyes (100% showed an outer retinal hole), with surrounding hyperreflective spot in the inner retinal layers in 4 eyes as early lesions which disappeared later on in the follow up period, the vitreoretinal interface was normal in 100% and RPE (Retinal Pigment Epithelium) were intact with lost inner border of RPE and loss of interdigitation zone (Figs. 3, 4, 5, 6).

All of the affected eyes were around emmetropia with a refractive error less than one diopter. It is worth mentioning that one of the patients with unilateral affection, the eye was affected (SE was-0.25 D) while the other unaffected eye was − 9.50D SE, The clinical data are shown in (Table 1).

**Table 1:** The clinical data at presentation and follow up of the patients

No. of cases	10 eyes
Time elapsed from exposure to evaluation	40% after 3 days 30% after 5 days 20% after 7 days 10% at the tenth day
Visual acuity on presentation	20% 2 eyes (0.2) 30% (0.3) 10% 0.4 20% 0.5 10% 0.7 10% 0.8
Visual acuity at the last follow up	50% to 0.9 20% to 1 20 to 0.8 10% to 0.4
Early symptoms	Central scotoma 100% Metamorphopsia 40%
Early findings	Amsler grid positive 100% Fundus white foveal lesion 30% Fundus autofluorescence (hyporeflective spot) 40% FA normal in 100% OCT outer retinal disruption 100%
Findings at Last follow-up	Amsler grid 30% OCT revealed an outer retinal layer hole in 80% Significant healing 20%

**Fig. 7:**
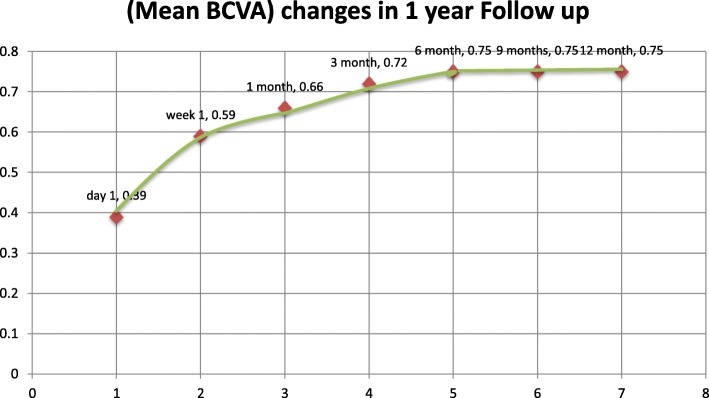
Shows a graph demonstrating the improvement of the mean BCVA in the 1 year follow up of solar maculopathy

Follow up continued for 1 year, at 1 week, 1 month, 3 months, 6 months, 9 months and 1 year after exposure. The VA started to improve 1 to 2 weeks after sun exposure, improvement continued to reach the maximum at 6 months and then reached a plateau level (Fig. 7), five eyes (50%) improved to 0.9, two eyes (20%) improved to 1, two eyes (20%) improved to 0.8 and one eye (10%) showed one line improvement to be 0.4from 0.3.

**Fig. 8:**
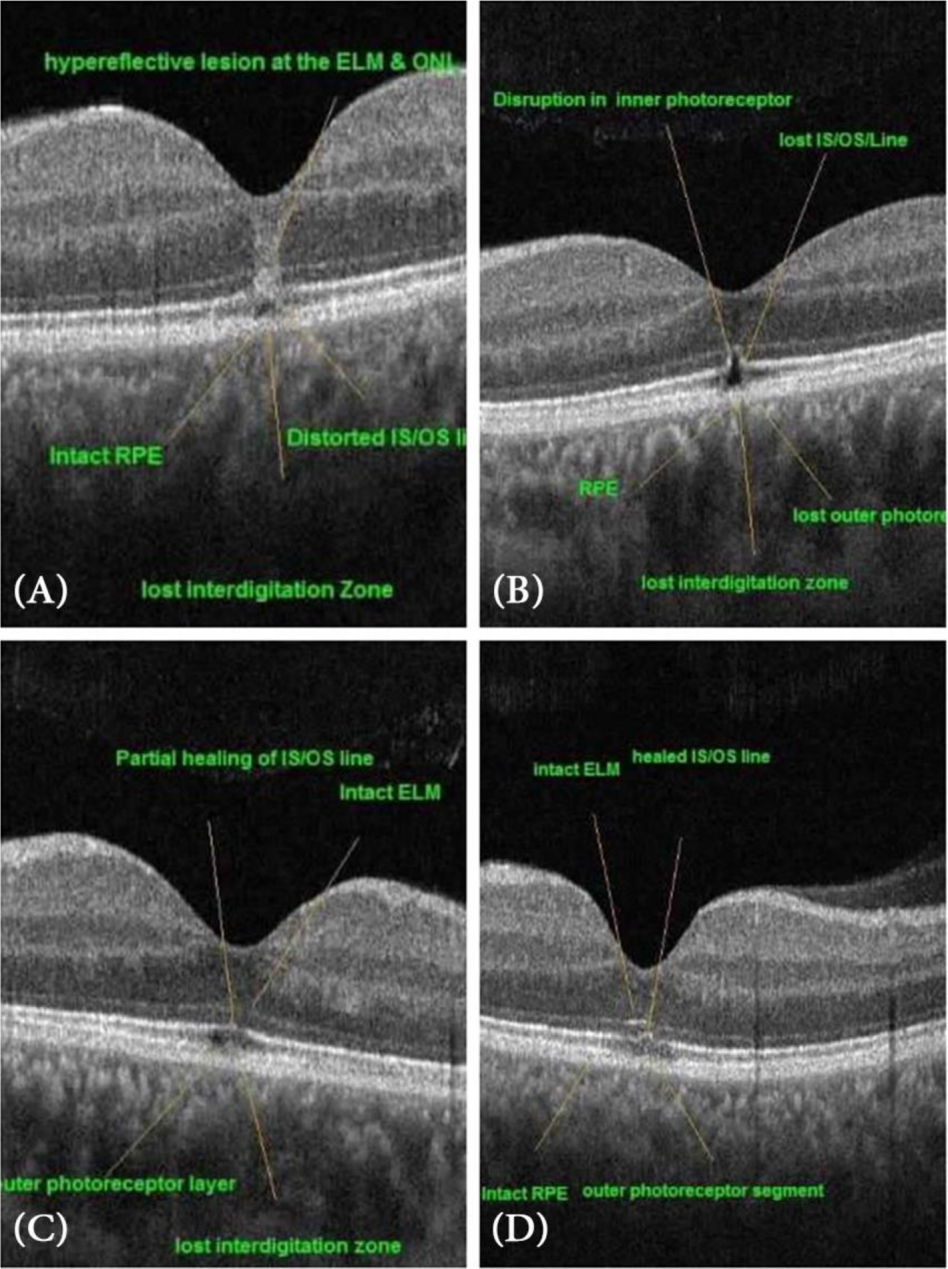
OCT images of the fovea in 1 year follow up, **a** shows the fovea after 1 week exposure demonstating a hypereflective band at the ELM (external limiting membrane) and ONL (outer nuclear layer) **b** shows a manifest outer retinal hole with disrupted photoreceptor and IS/OS line (inner segment/outer photoreceptor segment line) at 1 month, **c** shows 6 months OCT image showing partial healing of IS/OS line, **d** shows OCT image at 12 months with healed IS/OS line

While the vision improved in all cases by 100%, one eye (10%) showed the persistence of major vision affection 0.4 (improved one line only from 0.3). This eye showed a persistent outer retinal hole with disturbed ellipsoid zone and External limiting membrane as well. The outer retinal hole (ellipsoid and interdigitation zones disruption) persisted after 6 months up to 1 year in 8 eyes (80%) (Figs. 3, 4). While one patient with 2 eyes showed significant outer retinal healing after 1 year (Fig. 8).

## Discussion

Incident light on the retina transmitted through ocular media to the retinal pigment epithelium and photoreceptors within the retina. Ultraviolet (UV) radiation comprises invisible high energy rays from the sun that lie just beyond the violet/blue end of the visible spectrum. Most of the UV radiation is absorbed by the anterior structures of the eye, although some of it does reach the light-sensitive retina. The cornea is responsible for absorbing and filtering the shortest and thus most energetic UV radiation (UV-C < 280 nm). The young lens primary absorbs UV-A, whereas with age, whereas an older lens has the capability to impede UV-B (280 to 315 nm) transmission [7, 8].

Solar radiation is absorbed directly from the RPE. This damage leads to the reduced lipofuscin content of the RPE cells, which results from the disruption of the outer segments-to-RPE interdigitation [9].

Our patient age profile included children and young adults (range 9-27 years) which indicate that younger age patients are more prone to the harmful effect of the sun rays. This can be explained by the role of the lens, which becomes more protective with ageing [6]. Another compounding cause might be that the young people especially the children are more prone to sun gazing and they are also careless about using the proper eye protection or spectacles during direct sunlight viewing [10, 11]. While in previously reported studies, the age group was older, as the main category of patients were those suffering from psychiatric illnesses [11, 12].

Wu and colleagues reported the, UV radiation causes chemical damage by various mechanisms through free radical production and oxygen-dependent toxicity. The outer retinal layers of the macula are most vulnerable to photochemical damage. The main ultraviolet induced retinal lesions seem to be RPE cell disruption and photoreceptor outer segment damage [1, 13, 14].

In this study, the fundus picture shows absent foveal reflex and granular appearance of the fovea in all cases with a white small foveal lesion in 30% of cases. While a study done by Ault Jain et al., [15] stated the presence of significant foveal pathology in each of the 21 eyes (11 patients) included in their studies. In this study, Fundus autofluorescence shows a hypo-reflective area in 40% of cases, which may attributed to focal depletion of lipofuscin pigments in the photoreceptor layer [16].

Fluorescein angiography was normal in all cases (100%) in this study, this agreed with the case reported by Nakamura [17] in opposite to Ault Jain study as fluorescein angiography identified classic window defects in 19 eyes (10 of 11 patients) [15].

Solar retinopathy was first described using time domain OCT by Bechmann and colleagues [18] where they reported a hyper-reflective area at the fovea and affection of all retinal layers. Acute changes seen on OCT from solar retinopathy have been described to affect mainly the outer photoreceptor segments and may even resolve with time [14, 15].

In this study, The OCT shows an outer retinal layers disruption, a well-defined outer retinal hypo-reflective space interrupting the inner high reflective layer in 100% of our cases. This inner high reflective layer mostly corresponds to the junction between the inner and outer segments of the photoreceptors, while in the study of Ault [15] showed that optical coherence tomography demonstrated foveal atrophy associated with a characteristic lesion at the level of the inner and outer segment junction of the photoreceptors in all 21 affected eyes. But they mostly reported the findings late after a long time of sun exposure. And in the case reported by Ya-Hsin Kung [19], there was outer and inner retinal disruption. And this agreed with the case report mentioned by Suhr, [20] who found the obliteration of a portion of the retinal pigmented epithelium showing a pseudocystic like appearance. Actually, the occurrence of the inner retinal disruption in previous studies mostly due to chronic solar maculopathy associated with retinal atrophy and degenerations and this may occur with our cases with proceeding time [21].

The visual acuity improvement started after 7 to 10 days, this agreed with previous studies [22, 23] In all cases, an improvement in vision developed gradually until 6 months after exposure, then the vision became stationary and did not deteriorate and did not show further improvement. Although OCT shows an outer retinal hole disruption in 80% of cases and no significant macular abnormalities in 20% (this case whose eyes regained VA of 1), The persistent foveal changes in solar maculopathy is variable, many studies reported persistent foveal damage after 6 months as told by Wong [24] and Doyle [6] who reported a pseudohole in 7 eyes in a duration ranged from 3 to 12 months. While Kallmark [25] reported variable OCT changes after 1 year: 6 cases (40%) had mild disturbances, whereas 9 (60%) had none; 2 (13%) displayed RPE disturbance. In opposite to Awan [26] who mentioned that there are no macular changes after 6 months.

Amsler grid was positive for metamorphopsia and central scotoma in 30% of cases after 1 year although a good vision was gained, this condition is agreed with cases mentioned before, as the return of good visual acuity may occur, but some patients continue to experience visual deficiencies such as a small persistent central or paracentral scotoma due to chronic solar damage to the photoreceptors [27].

It was previously mentioned that the subjective improvement in VA occurred despite a persistent small hole in the ellipsoid zone detected by OCT even after 50 years after exposure [28].

In our study, 2 eyes (20%) BCVA improved to 1, and this improvement coincides with the complete healing of the outer retinal hole, that fact human photoreceptors cannot regenerate may be opposed by the hypothesis that the photoreceptors can regenerate if their nuclei are not damaged [29, 30]. The OCT images showed a hypereflective band in the inner retinal layers above the outer retinal hole in the first 2 weeks which disappeared later on, this hypereflective band was mentioned and postulated as some sort of inflammation [31].

The discrepancy between the visual acuity and the persistent outer retinal lesion in OCT is confusing, while others reported the same difference and claimed that the visual symptoms may not always correlate with physical findings [30, 32]. Roberts et al. [33] agreed our results as they found that the visual acuity was regained despite the persistent disruption in the ellipsoid zone and interdigitaion zone.

The improvement in vision may be related to the early healing of the external limiting membrane (ELM) before the ellipsoid zone and the interdigitation zone. This correlates with the same conclusion in other studies related to the integrity of the ELM to the improvement of VA in other macular diseases [34, 35].

One eye showed a persistent visual impairment at the end of 1 year follow up, mostly this attributed to the susceptibility of RPE affection, with secondary photoreceptors affection. Wu [1] reported that there are two classes of photochemical damage; class1 photochemical damage is mediated by the photoreceptors, while class 2 photochemical damage is generally confined to the RPE and may lead to secondary photoreceptor outer segment damage. It is worth to document that ELM did not heal in this case untill the end of the follow-up year. Actually, further longitudinal studies of these cases are needed, which show a late healing of the ellipsoid zone as the healing may take a longer time to occur. The role of ELM should be further investigated in the prognosis of solar maculopathy.

## Conclusion

Solar maculopathy occurs due to direct sun rays exposure, causing a diminution of visual acuity, with a good prognosis. OCT offers a definite diagnosis. Visual Acuity improves gradually till the sixth month; then it becomes stationary. The OCT pathology may still persist despite a good vision have gained. Young age might pose as a risk factor for affection. Further studies demonstrating the role of the external limiting membrane in prognosis are demanded.

## Data Availability

The datasets used and/or analyzed during the current study are available from the corresponding author on reasonable request.

## Abbreviations

BCVA: Best corrected visual acuity
D: Diopter
ELM: External limiting membrane
FA: Fluorescein angiography
FAF: Fundus autofluorescence
nm: Nanometer
RPE: Retinal pigment epithelium
SD-OCT: Spectral domain ocular coherence tomography
SE: Spherical equivalent
UV: Ultraviolet
VA: Visual acuity
